# Comparative genomics reveals high prophage diversity and horizontal gene transfer of effectors and phage defence systems in the Pseudomonas syringae complex

**DOI:** 10.1099/mgen.0.001711

**Published:** 2026-05-18

**Authors:** Dominique Holtappels, George E.J. Rickus, Túlio Morgan, Rafael R. de Rezende, Britt Koskella, Poliane Alfenas-Zerbini

**Affiliations:** 1 Plant Pathology and Plant-Microbe Biology section, School of Integrative Plant Science, Cornell University, Cornell AgriTech, Geneva, NY, USA; 2 Department of Integrative Biology, University of California, Berkeley, CA, USA; 3 Departamento de Microbiologia, Instituto de Biotecnologia Aplicada à Agropecuária, Viçosa 36570-900, MG, Brazil; 4 Chan-Zuckerberg Biohub, San Francisco, CA, USA

**Keywords:** accessory genome, horizontal gene transfer, pathogen adaptation, *Pseudomonas syringae*, Prophage

## Abstract

The mobilome, defined as the collection of mobile genetic elements within a bacterial genome, plays a role in the adaptation of bacteria to abiotic and biotic drivers. In particular, prophages have been reported to contribute to bacterial resistance to virulent bacteriophages, to competitive interactions among bacterial hosts within microbial communities and to pathogenicity and virulence. It is, therefore, critical to better understand the role of prophages in distributing genes and functions within and among bacterial species to predict how bacteria adapt to their biotic environment. *Pseudomonas syringae* offers an ideal study system to ask these questions, both because of its broad range of lifestyles (spanning from environmental growth to plant pathogens) and its high intraspecies diversity. To examine the role of prophages in this species complex, we compared 587 genomes available from public databases and annotated the defence mechanisms, effectors and prophages in the genomes. We found that this species complex has an elaborate phage pandefensome consisting of 139 defence mechanisms. Assessing taxonomical signatures of the observed prophages uncovered broad differences in the types and numbers of genes encoded by different phage families, emphasizing how the evolutionary advantages conferred to hosts can depend on the prophage composition and offering insight into how these genes might disperse within a community. Our study highlights the intimate association of specific phage families with their hosts and their potential role in shaping key ecological traits of these important species.

**Impact Statement**

The bacterial accessory genome, including the mobilome and prophages, plays a critical role in shaping bacterial adaptation to abiotic and biotic drivers. These prophages are widespread across bacterial taxa and likely maintained because of their evolutionary advantage. Our ability to predict how a bacterial population will evolve requires a better understanding of where key functional traits arise. To address this question, we assessed prophage-encoded phage defences and effectors across *Pseudomonas syringae*. We show that prophages carrying these genes belong to specific phage taxa with differences in the types of genes encoded. This emphasizes the evolutionary advantage of these prophages, offering a framework to uncover how these genes disperse within microbial communities and their role in pathogen evolution.

## Data Summary

The genomes analysed in this study are provided in Table S1.

## Introduction

Bacteria are continuously under evolutionary pressures from abiotic and biotic interactions within a given environment [1,2]. To respond to these complex dynamics, bacteria must evolve rapidly through high mutation rates, rapid generation times and remarkable genome plasticity from acquiring mobile genetic elements like phages and plasmids [3,4]. In bacterial pathogens, the acquisition of virulence genes embedded in pathogenicity islands and viral vectors can lead to host range expansion and key changes in virulence [5]. Host-associated bacteria face selection pressure from their eukaryotic hosts, bacterial competitors and their bacteriophages [6,7]. As lytic phages lyse and kill bacterial cells, they impose strong evolutionary pressure on susceptible bacterial populations, resulting in diverse resistance strategies that continually reshape who is infecting whom [8]. Recent advances show that horizontal gene transfer can drive the distribution of phage defence mechanisms [9,10], suggesting the mobilome is critical for bacterial adaptation in host interactions and viral resistance [2].

As part of the mobilome, prophages and viral satellites, among other types of mobile genetic elements, can act as carriers for the dispersal of genetic information between bacteria in a community. Often, these genes offer the host an evolutionary advantage, referred to as lysogenic conversion [11]. These genes participate in metabolic pathways, influence the outcome of biotic interactions within the community [12,13] or shape the bacterium–host interaction [11]. Examples include auxiliary metabolic genes, such as those involved in carbohydrate metabolism and two-component transporters for the uptake of specific compounds [14], and antimicrobial resistance [15]. These genomic parasites not only modulate bacterium–bacterium interactions but also shape the outcome of phage–bacterium interactions themselves. As prophages are vulnerable to other superinfecting phages, they have evolved to carry mechanisms that aid in surviving phage predation, referred to as superinfection exclusion mechanisms (as reviewed elsewhere [16]). Alongside these specialized mechanisms, prophages can also encode restriction enzymes [17,18], CRISPR Cas phage defence mechanisms [19], and abortive infection mechanisms (Abi) like BstA [20]. Lastly, prophages are capable of drastically shaping bacterium–host interactions through the incorporation of genes involved in the pathogenesis of the host or the modulation of the bacterium–host interaction. For example, *Vibrio cholerae* depends on the presence of prophages encoding the cholera toxin for its pathogenicity [21]. Further prophage-encoded proteins underlying pathogenesis include the shiga toxin in Shiga toxin-producing *Escherichia coli*, the diphtheria toxin in *Corynebacterium diphtheriae,* the botulinum toxin in *Clostridium botulinum* and the SopE effector in *Salmonella enterica*, among others [22,23]. In *Staphylococcus aureus*, beta-haemolysin-converting prophages even encode genetic clusters that aid the bacterial host to evade the immune system of the eukaryotic host and are associated with disease severity in chronic inflammatory diseases like chronic rhinosinusitis [22,24]. These examples illustrate the intricate interaction between bacteria and their genomic parasites and how they shape the adaptability of their host, ranging from abiotic to biotic interactions, further illustrating the intricate interaction between bacteria and their phages.

Open questions remain about the distribution and phylogenetic conservation of mobile genetic elements encoding bacterial virulence genes and phage defence mechanisms. *Pseudomonas syringae* offers an ideal system to address these questions. This diverse species complex consists of 13 different phylogroups with high interspecific diversity [25]. It includes both highly specialized phytopathogens causing disease in a myriad of commercially and environmentally relevant plants, as well as isolates that are non-host associated. Primary phylogroups (PG1, 2, 3, 4, 5, 6 and 10) were isolated directly from diseased hosts, while isolates belonging to the secondary phylogroups (PG7, 9, 11 and 13) were isolated from environmental sources (i.e. soil and water) [26,28]. The mobilome of this species complex has been shown to play an intricate role in the adaptation of *P. syringae* to its abiotic and host environment. Plasmids, for example, are described to aid in the resistance against xenobiotics (i.e. copper chemicals and antibiotics, such as streptomycin) across different isolates [29], and the type III effector proteins (key pathogenicity drivers) are often found to be encoded on pathogenicity islands and plasmids [30,33]. These effector proteins are injected directly into the plant cell, impacting both pathogen and effector-triggered immunity responses as well as permeabilizing the cell wall of plant cells [34]. Recent data demonstrated that *P. syringae* pv. *morsprunorum* was found to encode HopAR1 effectors embedded in prophage sequences that were found to excise and circularize, leading to successful transfer from one bacterium to another [35]. Although data such as these clearly demonstrate the potential importance of prophages in the pathogenesis of *P. syringae*, there has been relatively little work on whether and how prophages carry phage defence mechanisms.

To address this knowledge gap, we characterized the phage defensome, effectorome and prophage-like elements encoded by 587 publicly available *P. syringae* genomes. We specifically evaluated horizontal gene transfer likelihood for defence mechanisms, established prophage networks between phylogroups and identified specific taxa of prophages that carry both phage defences and effector genes.

## Methods

### Data acquisition, quality control and phylogenetic analysis

A dataset of 630 draft genomes of the *P. syringae* species complex was formed by extracting all strains available (in January 2023) from the National Center for Biotechnology Information (NCBI) taxonomy database using the taxonomic classifier ‘*Pseudomonas syringae*’ (Table S1, available in the online Supplementary Material). As this approach was biassed towards PG1, PG2, PG3, PG7, PG9 and PG10, resulting in an underrepresentation of PG5 and PG6, we supplemented our dataset, based on Dillon *et al.* [30]. The dataset was curated for doubles, and the quality of each strain’s genome was assessed using BUSCO (v5.4.7), and strains with a score of at least 0.95 were used in all further analyses [36]. All genomes included in this study are summarized in Table S1. A phylogenetic analysis on the soft-core genome of the different isolates was performed by means of PPanGGOLin (v1.2.105) [37] together with FastTree with 1,000 bootstraps and default parameters as previously described [38,39].

### Annotation of bacterial genomes and analysis of the phage defensome and effectorome

The draft genomes were annotated with Prokka (v.1.14.5) for an ORF prediction [40]. The FASTA amino acid files were mined for phage defence mechanisms by means of DefenseFinder (v.1.0.9) [41] with default parameters. The output files were compiled, and different defence mechanisms were summarized per draft genome. Similarly, the genome faa files were mined for the presence of effector genes by means of phmmer (*e*-value <1E−60) using the database as described by Dillon *et al*. [30]. Statistical analyses and data visualization were performed by means of the JMP Pro16 software [42]. An absence/presence matrix was constructed, and a hierarchical clustering was performed by means of JMP Pro16 [42]. In short, the hierarchical agglomerative clustering method within JMP was used to identify similarity between the patterns in the data using Ward’s method based on the distance between two clusters as the ANOVA sum of squares between the two clusters summed over all the variables. Across generations, the sum of squares is minimized over all partitions [43]. The similarity of defensomes between phylogroups was evaluated by an analysis of similarity (ANOSIM) using the VEGAN package in R [44].

### *In silico* analysis of horizontal gene transfer

A RecPD (v0.0.0.9000) analysis was used to calculate the degree to which every defence mechanism was transferred through horizontal gene transfer in the *P. syringae* species complex [45]. The phylogenetic tree of the *P. syringae* species complex and the absence presence matrix generated from DefenseFinder were provided to the RecPD program as input. The nRecPD statistic for each defence mechanism was calculated by dividing the total branch length in which the defence mechanism was transferred vertically by the total length of branches of the tree rooted at the defence mechanism’s most recent common ancestor [41,45]. All defence mechanisms with a nRecPD score lower than 0.25 were used in all subsequent analyses of horizontal gene transfer within the *P. syringae* species complex.

To this end, the genomic islands as determined by the PPanGGOLin (v1.2.105) analysis were mined for the defence mechanisms with the highest probability of being dispersed by horizontal gene transfer. A blast database containing a random instance of every defence mechanism with a nRecPD score lower than 0.25 was constructed using the makeblastdb application (v2.14.0) with default parameters for nucleotide sequences. All DNA sequences of each defence mechanism found in the *P. syringae* species complex were obtained through a blastn analysis of these genomic islands using this database of defence mechanisms, an *e*-value of 1E−6 and default values for all other parameters [37,38].

### Prophage analysis

The *Pseudomonas* genome sequences were submitted to PHASTER [46] via the API to detect prophage-like sequences. Since many *Pseudomonas* genome assemblies were fragmented into multiple separated fasta sequences, we analysed whether the questionable and incomplete prophages identified by PHASTER were near the contig edges. In this scenario, the prophage-like sequences could be artificially fragmented, hindering the completeness estimated by PHASTER. Following this, the putative questionable or incomplete prophages occurring in 1,000 bp or less from contig edges and sizes less than 10 kb were considered as possible artificially fragmented sequences.

All identified prophage-like sequences were submitted to Prokka v.1.14.5 [40] for ORF prediction (parameters: --kingdom Viruses --gcode 11). The heatmaps showing prophage occurrence were plotted within a *Pseudomonas* soft core genes phylogenomics. The plot was generated using the ‘ggtree’ package [47] in the R environment (R Core Team, 2013).

The gene-sharing networks of the prophage-like sequences were conducted using vConTACT2 [48] (parameters: --rel-mode ‘Diamond’ --db ‘None’ --pcs-mode MCL --vcs-mode ClusterONE). Cytoscape v.3.8.2 was used for networking visualization. Node colours and shapes were set based on *Pseudomonas* phylogroups and prophage sequence completeness, respectively.

We used VIRIDIC [49] to obtain an overview of whole-sequence similarity between prophage-like sequences. VIRIDIC uses local alignments (blastn) to calculate nucleotide-based intergenomic similarities and sequence clustering in ‘species_cluster’, as long as they share sufficient intergenomic similarities (default threshold of 95% for species clustering). The plots were generated using the ‘circlize’ package [50] in the R environment.

Similarly, the prophages that were annotated to encode effector genes and phage defence mechanisms were taxonomically classified by means of VipTree [51], and the intergenomic similarity was calculated with VIRIDIC and visualized with the cluster heatmap package from Seaborn (v0.13.0) [52].

## Results

### The *P. syringae* species complex represents a diverse panel of isolates with elaborate phage defensomes

**Fig. 1.**
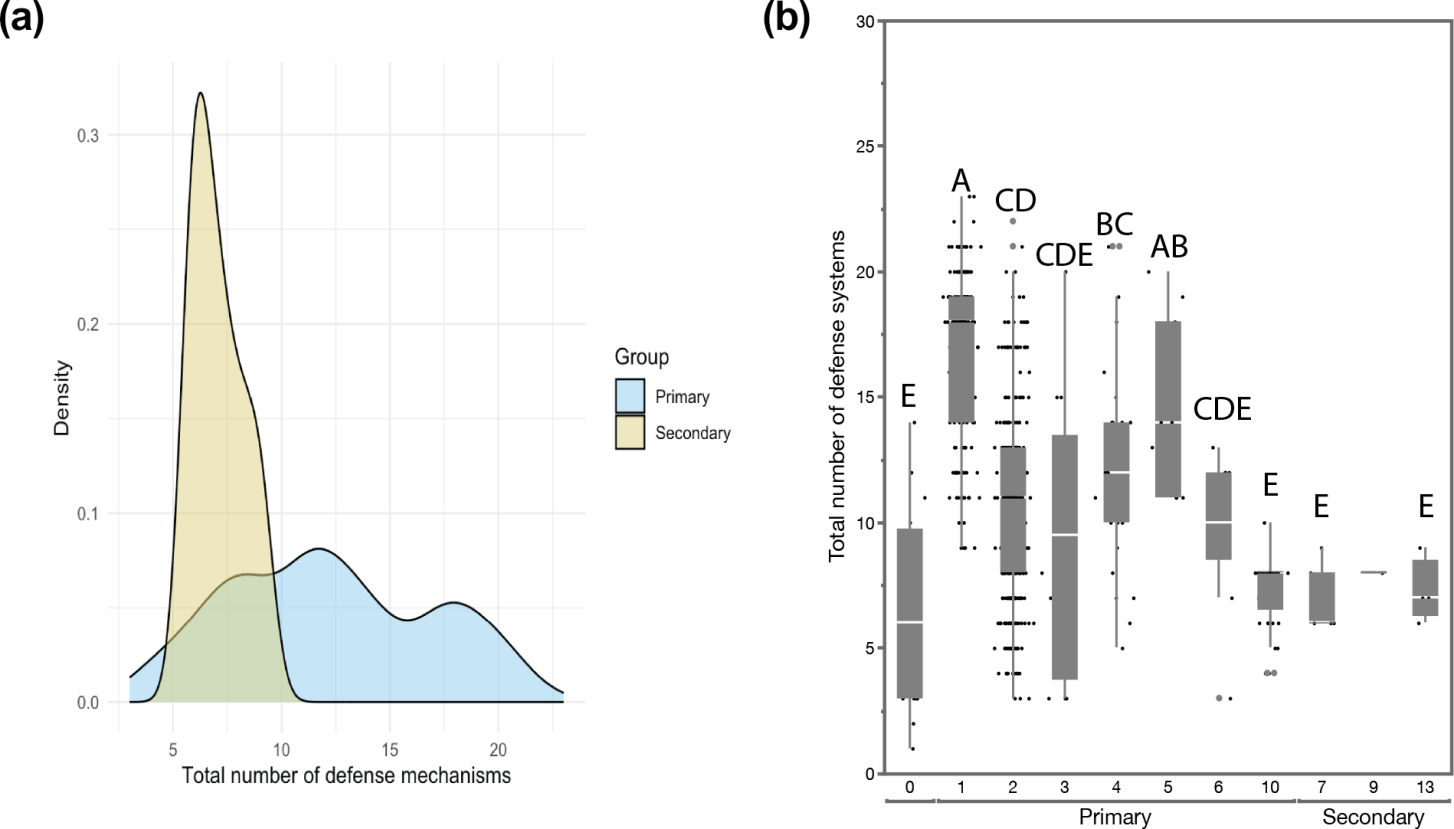
Number of phage defence mechanisms encoded in the *P. syringae* species complex. (a) Distribution of primary (blue) and secondary (yellow) phylogroups and the total number of phage defence mechanisms encoded with significantly more phage defence mechanisms encoded in the primary phylogroups (Student’s t-test, *P*-value=0.0002). (b) Quantile box plots of the total number of phage defence mechanisms per individual phylogroup. Based on a Tukey–Kramer test with corrections for multiple comparisons, four groups of significance were identified with phylogroups one on average the highest number of phage defence mechanisms per genome, as summarized using connecting letters indicating the groups of significance (A, B, C, D and E, *P*-value <0.05).

We extracted over 680 assemblies of *P. syringae* genomes from NCBI, corrected for duplicates and evaluated their completeness by means of a BUSCO analysis. As a result, we narrowed the dataset down to 587 genome assemblies with a BUSCO score greater than 0.95. These genome assemblies were used to calculate the phylogenetic relationship between the different isolates (Fig. S1). An overview of the different genomes analysed in this study is provided in Table S1 and includes information on the phylogroup as determined by Dillon *et al.* [30]. We screened the genomes for over 60 different types and 150 subtypes of phage defence mechanisms, as currently reported in literature (Fig. 1). We found that throughout the species complex of *P. syringae*, isolates encoded, on average, 12 defence mechanisms in their genomes. In terms of the number of defence mechanisms that were encoded by the different phylogroups (Fig. 1a), we looked at the distinction between primary and secondary phylogroups as previously established [28,30]. Due to substantial sample size imbalance between primary and secondary phylogroups, we employed non-parametric statistical methods. We used the exact Wilcoxon–Mann–Whitney test to compare defence mechanism counts between phylogroups. Effect sizes were calculated using both Cohen’s *d* and Cliff’s delta to assess biological significance. Bootstrap resampling (10,000 iterations) was performed to generate robust confidence intervals for mean differences. To validate our findings, given the sample size imbalance, we conducted additional subsampling analysis by repeatedly sampling 12 genomes from the larger group and comparing them to the smaller group (1,000 iterations). Defence mechanisms differed significantly between phylogroups (exact Wilcoxon–Mann–Whitney test: *Z*=4.07, *P*=1.08×10⁻⁵). The primary phylogroups encoded substantially more defence mechanisms per genome (mean±sd: 12.3±4.9, median: 12, range: 3–34) compared to the secondary phylogroups (7.1±1.2, median: 7, range: 6–9). This represents a mean difference of 5.2 defence mechanisms per genome, with a large effect size [Cohen’s *d*=1.09, Cliff’s delta=0.69 (95% CI: 0.57–0.77)]. Bootstrap analysis confirmed the robustness of this difference (95% CI: 4.46–6.01). Subsampling validation showed that 88.8% of equal-sized comparisons (*n*=12 each) remained statistically significant, confirming that our findings are not artefacts of sample size imbalance. When looking at the individual phylogroups, we identified that within the primary phylogroups, there are three levels of defence: highly elevated in phylogroup 1 (17 mechanisms on average) and 5 (14 mechanisms on average), somewhat elevated in phylogroups 2, 3, 4 and 6 (~11 mechanisms on average), and a low number of defence systems in phylogroup 10 (7 mechanisms on average; groups A, B, C, D and E – Tukey–Kramer test with correction for multiple comparison, *P*-value <0.05, *P*-value between group A and E is <0.0001).

**Fig. 2.**
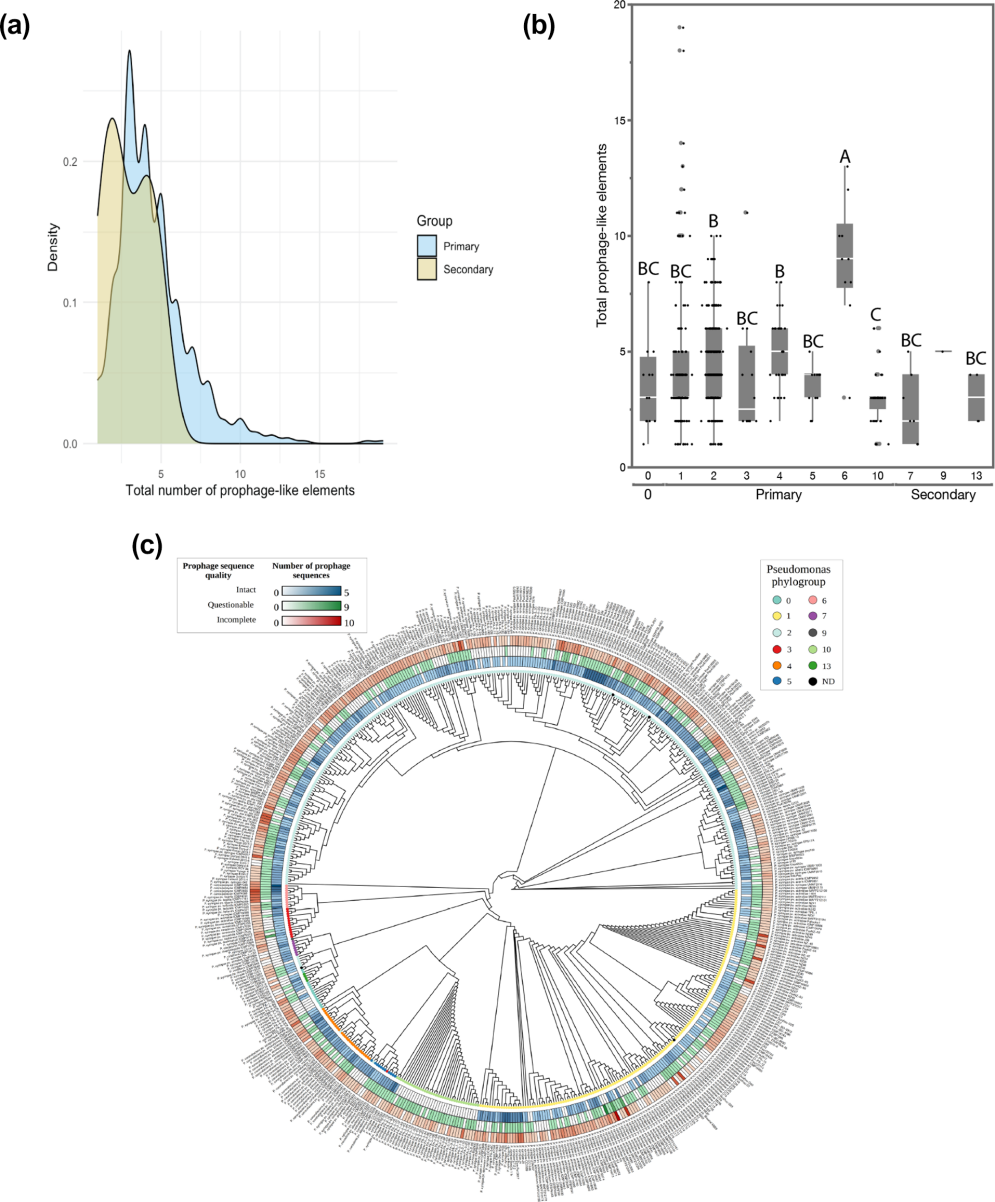
Gene sharing network between the different prophage genomes annotated in the *P. syringae* species complex. The nodes are coloured along the phylogroup (PG0, teal; PG1, yellow; PG2, light blue; PG3, red; PG4, orange; PG5, blue; PG6, pink; PG7, purple; PG9, grey; PG10, light green; PG13, dark green). Every connection represents a similarity to another predicted viral sequence. The different clusters are outlined and numbered (I, II, III, IV, V, VI, VII).

The total pandefensome of *P. syringae* consisted of 139 types of defence mechanisms. Based on our annotations, the Septu mechanism was the most abundant in the *P. syringae* pandefensome with 911 annotated mechanisms, followed by Gabija (*n*=546) and type IV and type II restriction modification systems (*n*=529 and *n*=465, respectively). The least abundant mechanisms were related to other abortive infection mechanisms (Abi), CRISPR-Cas and BREX (*n*=1) (Figs 2 and S2). Additionally, we looked at the effect of genome quality (draft genomes assembled from short reads and complete genomes assembled from a hybrid between short and long reads), and we have not found a significant difference in the total number of phage defence mechanisms annotated in the genomes (ANOVA, *P*-value=0.2155, Fig. S1).

Moving from the phylogroup level to the individual genome level, we observed that certain isolates encoded multiple copies of the same class of defence mechanism. We found that there were up to three copies of PfiAT, RM type I, RosmerTA and Shedu and four copies of Gabija, Lamassu, RM type II and RM type IV incorporated in the genomes of *P. syringae* isolates. In the case of Septu, up to five copies were annotated (BS2900, H2a, J6, MAFF 212171, PA-5–7A, PS25, SeraOz_1_1 r and USA007 all isolates within PG1).

### Overall patterns in the *P. syringae* phage defensome are phylogenetically conserved with clear evidence of horizontal gene transfer for certain mechanisms

To investigate the phylogenetic conservation of overall patterns in the phage defensome and of *P. syringae*, we summarized all the annotated defence mechanisms according to the individual isolate and clustered the strains based on the patterns of the absence/presence of a specific mechanism (Fig. S5). Then, to evaluate the similarity of the patterns observed in the different defensomes within and between phylogroups based on the absence/presence of a specific mechanism, we performed an ANOSIM. These analyses uncovered differences in the defensomes based on phylogroup (*R*=0.274 and *P*-value=0.001), suggesting a higher similarity of the defensome within phylogroups than among phylogroups. Despite the phylogenetic relation in the defensome profiles, we observed clear randomness in the profiles within the clusters, hinting towards the horizontal dispersal of certain phage defence systems. To test the likelihood that a specific defence mechanism is acquired horizontally rather than vertically, we calculated the normalized recombination phylogenetic diversity statistic (nRecPD) for each of the defence mechanisms (to ensure statistical rigour). The closer the nRecPD value is to zero, the more likely it is acquired horizontally rather than vertically through phylogeny [34]. Fig. S6 summarizes the individual statistics for each defence mechanism. Based on our calculations, 67 out of the 139 mechanisms (48%) were found to be likely distributed in the *P. syringae* species complex by means of horizontal gene transfer (nRecPD <0.35), highlighting horizontal distribution as a dominant driver of diversity in this pathosystem.

### The *P. syringae* species complex carries a wide diversity of genomic parasites

The 587 high-quality bacterial genome assemblies were subjected to a prophage sequence search using PHASTER [35]. A total of 2,595 prophage-like sequences were detected in nearly all genomes. Most prophage-like sequences were incomplete (1,235/2,595=47.6%), followed by intact (860/2,595=33.1%) and questionable (500/2,595=19.3%) sequences. Among *P. syringae* genomes harbouring prophage-like sequences, the number of elements ranged from 1 to 19, with only 92 genomes (15.6%) free of intact prophages (putatively active phages).

We further evaluated whether assembly quality affected prophage annotation. Unlike phage defence mechanisms, we observed significant differences between draft genomes (short reads) and complete genomes (hybrid sequencing) for total prophage-like sequences, including both incomplete and intact prophages (ANOVA, *P*<0.001, Fig. S7). This suggests that assembly quality influences the number of prophage-like sequences annotated in genomes.

**Fig. 3.**
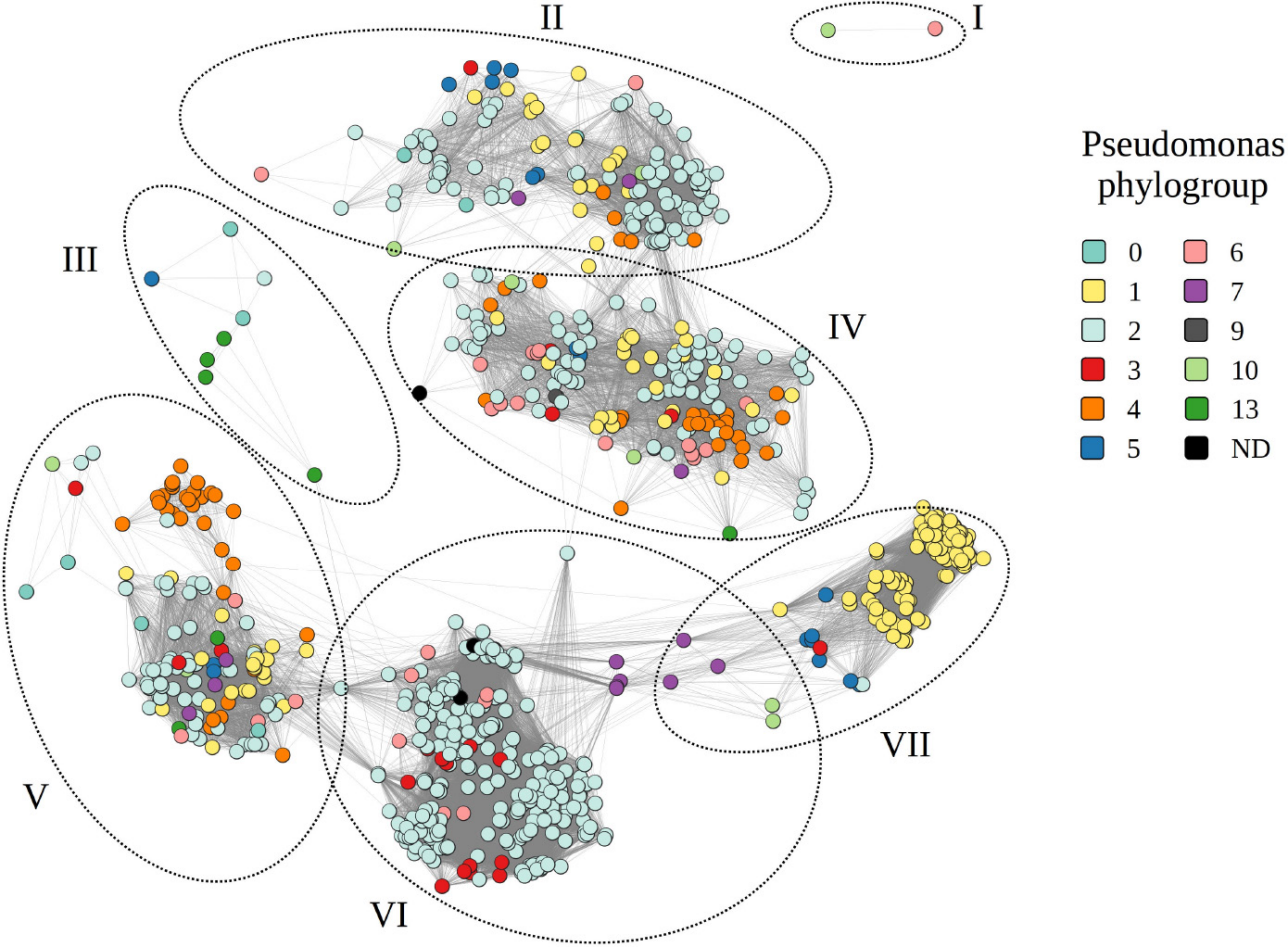
Number of prophage-like elements encoded in the *P. syringae* species complex. (a) Distribution of primary (blue) and secondary (yellow) phylogroups and the total number of prophage-like elements encoded with significantly more prophage-like elements encoded in the primary phylogroups (Student’s t-test, *P*-value=0.0017). (b) Quantile box plots of the total number of prophage-like elements per individual phylogroup. Based on a Tukey–Kramer test with corrections for multiple comparisons, three groups of significance were identified with phylogroups 6 on average the highest number of prophage-like elements per genome as summarized with connecting letters indicating the groups of significance (a, b and c). (c) Phylogenetic tree of 587 *Pseudomonas* isolates. The ‘ggtree’ package in the R environment was used for tree visualization ignoring branch lengths. Heatmaps were added as concentric rings at the tree tips, representing the quality and quantity of prophages detected in each *P. syringae* genome by PHASTER.

Similarly to the defence mechanisms, we observed a significant difference between the lifestyle (primary and secondary phylogroups) and the total number of prophage-like elements encoded in the genome (Student’s t-test, *P*-value=0.0017, Fig. 3a). Because the assembly quality significantly impacted the number of prophage-like elements annotated in the genomes, we tested whether this significant difference holds up only looking at high-quality assemblies. Indeed, based on a Student’s t-test, we found that primary phylogroups encoded a significantly higher number of prophage-like elements compared to the secondary phylogroups (*P*-value=0.0012) when only assessing complete genomes. Fig. 3c depicts the density of prophages within the phylogenetic tree of *P. syringae* isolates. In the case of the intact prophages, there is no significant difference (*P*-value of 0.22). Looking at the individual phylogroups, we found three groups of significance (significance level A, B and C – Tukey–Kramer test with corrections for multiple comparisons, *P*-value <0.05) and that phylogroup 6 and 4 carry on average the most genomic parasites (both complete and incomplete prophages), followed by phylogroups 2 and 3 (Tukey–Kramer test with corrections for multiple comparisons, *P*-value <0.05, Fig. 2b). Interestingly, phylogroup 1 contained on average the least number of genomic parasites in their genomes. Focusing on the intact prophages specifically, PG6 encodes the highest number of intact prophages, significantly more compared to PG2 and PG1 (Tukey–Kramer test with corrections for multiple comparisons, *P*-value, 0.05). The latter has the least number of intact prophages. Focusing only on high-quality assemblies, we found similar trends in the data with a significant difference between the number of intact prophages between PG1 and PG6 (Tukey–Kramer test with corrections for multiple comparisons, *P*-value<0.05). As PG1 encoded the largest number of defence mechanisms, we hypothesized that the number of genomic parasites declines with an increasing number of phage defence mechanisms. However, we found very weak correlations between the two variables for all phylogroups, suggesting that the number of defence mechanisms is a poor predictor for the number of prophage-like elements for all phylogroups (Fig. S8).

We employed a combination of vConTACT2 and VIRIDIC to access the sequence diversity of prophages. While vConTACT2 generates sequence clusters based on protein-sharing between phages, VIRIDIC uses local nucleotide alignments (blastn) to group sequences into species clusters and genus clusters according to user-defined thresholds of intergenomic similarities. Visualizing the intact prophages by means of a vConTACT2 analysis, we distinguished seven clusters (Fig. 2). The different nodes were strongly interconnected, depicting a high degree of similarity between the different genomes, especially between prophages in cluster II and cluster IV and clusters V, VI and VII. Prophages belonging to clusters II, IV and V consist of prophages from different phylogroups, highlighting the taxonomical relationship between prophages in these phylogroups. Clusters VI and VII, on the other hand, are more homogeneous as they primarily consist of prophages within phylogroup 2 and phylogroup 1, respectively. We also identified two clusters of phages with little similarity to the other clusters (clusters I and III). Fig. S9 shows a vConTACT2 network of all prophage-like elements. In total, VIRIDIC clustered the 2,595 prophage-like sequences in 1,282 species clusters, many containing only one sequence. The high sequence diversity was also evident by visualizing all pairwise comparisons between prophage-like elements, with few sequences sharing more than 65% of intergenomic similarities. Below this level, the prophage-like sequences are considered distantly related [24]. Overall, the prophage-like elements occurring in a given species cluster had a narrow host range, occurring in a specific *Pseudomonas* subspecies (Fig. S10).

### The role of genomic parasites in the establishment of the *P. syringae* phage defensome and effectorome

**Fig. 4.**
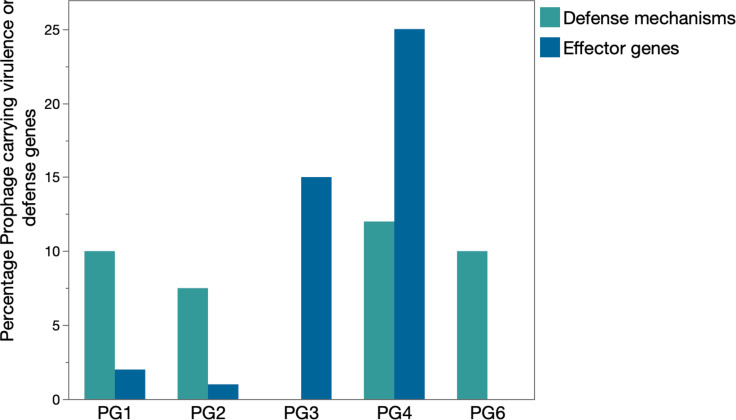
Percentages of intact prophages carrying a phage defence mechanism (teal) or an effector gene (blue) organized by the different phylogroups. While 5–12% of the intact prophages embedded within the genomes of PG1, PG2, PG4 and PG6 isolates carry at least one phage defence mechanism, up to 15% and 25% of the intact prophages within PG3 and PG4 encode at least one virulence gene, respectively.

Next, we mined all prophage-like elements for the presence of either phage defence mechanisms and/or type III effector genes, as previously reported [30]. To exclude as much bias as possible and ecological relevance, we separated the prophage-like elements into cryptic prophages and viral satellites based on the PHASTER output (which are typically not able to disperse on their own and are more likely to be computational artefacts) and intact prophages that encode all the genetic information necessary to excise from the host genome and enter a lytic replication cycle. Based on our dataset, we found that 7% of prophages encoded phage defences 64/860 and 3% encoded effector genes 28/860. Dividing the genomes according to phylogroup and focusing only on intact prophages, we found that there was a difference between the prophages embedded in the genomes of different phylogroups. Only a minor fraction of the prophages within PG1 and PG2 (2% and 1%, respectively) carry effector genes, while in the case of PG3 and PG4, these percentages reach 15% and 25%, respectively (Fig. 4). In the case of defence mechanisms, prophages within PG2 encode 7.5%, PG1 and PG3 10% and PG4 12% (Fig. 4). Strikingly, several of the prophage-encoded phage defence systems were characterized with low nRecPD values (<0.35; DRT8=0.0364–14% prophage-encoded, Pycsar=0.0744–8% prophage-encoded, PARIS-I=0.081–18% prophage encoded, BstA=0.132–6% prophage-encoded, DsrI=0.156–3% prophage-encoded and AbiL=0.217–14% prophage-encoded), confirming that these mechanisms are likely to be distributed through horizontal gene transfer. Yet, intact prophages only account for a minor percentage of the total abundance of these mechanisms. Notably, high-quality assemblies (complete genomes vs. draft genomes) tend to carry more prophage sequences. As such, we can hypothesize that as more high-quality genomes are becoming available and annotation algorithms further improve, in the near future, we will be able to have a clearer image of the genetic cargo encoded by these mobile genetic elements.

**Fig. 5.**
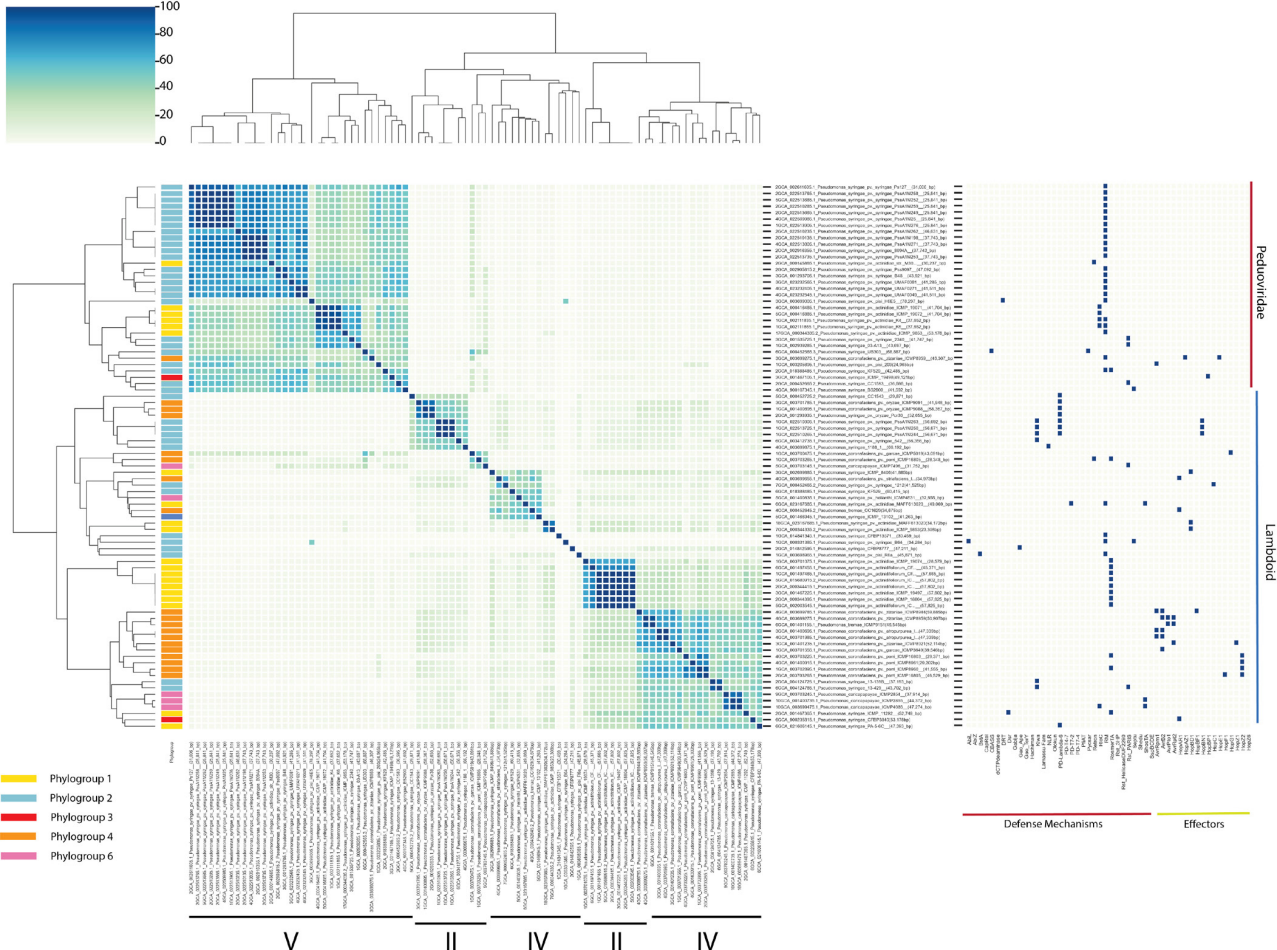
Taxonomic analysis of the prophage genomes as determined by a pairwise comparison of intergenomic similarity (VIRIDIC – left panel) and the absence/presence matrix of the different phage defence mechanisms and effector genes encoded by the prophage genomes (right panel). The different prophage genomes were clustered according to their genome similarity (100% in blue, 0% in light green). The phylogroups in which the prophage genomes were annotated are colour coded (PG1, yellow; PG2, light blue; PG3, red; PG4, orange; and PG6, pink). The prophages split into two main clusters: the *Peduoviridae* and the lambdoid phages as determined by a VipTree analysis. Within the lambdoid phages, two clusters can be distinguished. The different clusters as determined in the gene sharing network are depicted below (II, IV and V).

We compiled all the intact prophage genomes that encoded either phage defences, effector genes or both and performed a taxonomic analysis of the phages that carried them. Indeed, based on a Viptree and VIRIDIC analysis, we observed that the prophages clustered together into two main lineages, with one outlier (Fig S10 and S11). One of the main lineages represented a novel group of phages that cluster within the *Peduoviridae* family. The second lineage was related to other, previously observed lambdoid *P. syringae* phages, including *P. syringae* pv. *morsprunorum* phage MR15 [53], *P. syringae* pv. *actinidiae* phages psageK9 [54], PhiSA1 [55], PhiAH14a [56] and *P. syringae* pv. *tomato* phage Medea1 [57]. This latter clade split into two subgroups. As summarized in Fig. 5, the P2-like phages (*Peduoviridae*) mainly occur in isolates belonging to phylogroup 2 (76%, 25/33). In the case of the lambdoid phages, one subgroup occurred mainly in phylogroup 2 (50%), followed by phylogroup 4 (27%). The other subgroup was primarily associated with phylogroup 4 (41%) and phylogroup 1 (37%). Notably, within phylogroup 1 and phylogroup 2, the intergenomic similarity between prophages harboured in related strains approaches 100%, suggesting that these prophages are stably transmitted within lineages. However, most prophages share lower similarities, suggesting that these phages are different species (<95% intergenomic similarity) and are likely horizontally acquired.

**Fig. 6.**
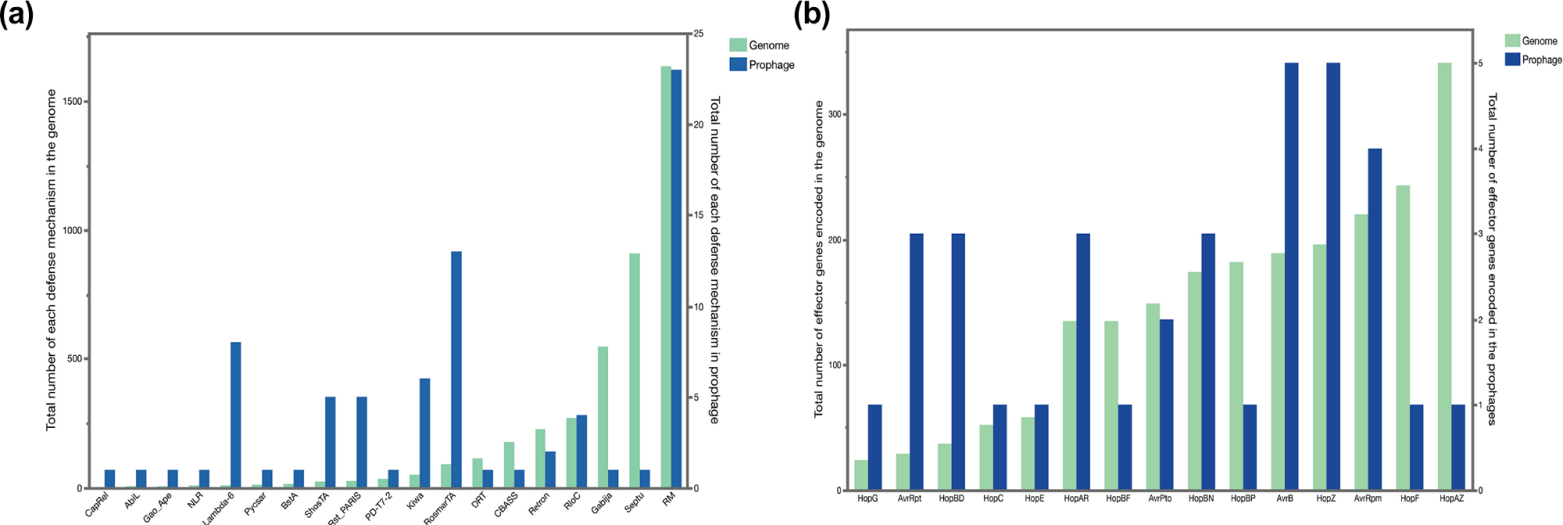
Total abundance of phage defences (a) and effector genes (b) encoded by the prophage genomes. The left *y*-axis depicts the abundance of the mechanisms in the genomes (teal), and the right *y*-axis graphs the total abundance in the prophage genomes (blue). Based on Wilcoxon signed rank tests, certain phage defences and effector genes were enriched in the prophage genomes (*P*-value <0.001).

The most abundant phage defences encoded within the prophage genomes were restriction modification systems and RosmerTA, potentially stabilizing the prophage as mobile genetic elements [58]. While the former is most abundant in the *Peduoviridae*, the latter is most prominent in the second clade. Overall, we found significant differences between the number of phage defences that were genomically or prophage encoded (Wilcoxon signed rank test; *P*-value <0.001, Fig. 6a). Based on contingency tables, we observed that the defence mechanism PD-Lambda 6 was enriched in the prophage genomes as 1.3% (*n*=10/587) of genomes encoded the mechanisms vs. 12% of prophages (8/66) (*P*-value <0.0001).

Regarding effector genes in our dataset, we observed that lambdoid phages were the ones primarily carrying effector genes, with only a minority of the *Peduoviridae* annotated to contain effector genes (3/33). We found that *avrB*, *hopZ* and *avrRpm* are relatively abundant within intact prophage genomes. Other effector genes that are common in *P. syringae*, like *hopAZ* and *hopF*, are less abundantly present in the prophage genomes. Again, based on a Wilcoxon signed rank test, we found a significant difference between the types of effectors observed in prophage compared to the genomic background of *P. syringae* (*P*-value <0.001, Fig. 6b). However, we did not observe a significant enrichment of effector genes in the prophage genomes based on contingency tables.

## Discussion

Like all organisms on Earth, bacterial populations are constantly evolving in response to the abiotic selection pressures. For bacterial pathogens, these pressures are particularly intense, including those imposed by phages and host immune responses. The mobilome offers a rapid and effective mechanism to both spread important traits within a community and increase evolutionary flexibility. We leveraged the well-studied and highly sequenced *P. syringae* species complex to determine the importance of prophages in shaping bacterial responses to viral and host-associated selection pressures using a comparative genomics approach.

In our study, we focused on high-quality genomes of the *P. syringae* species complex, leading to a high representation of phylogroups 1 and 2. We found that *P. syringae* isolates harbour elaborate phage defensomes. While the pandefensome of the species complex consists of 139 different defence mechanisms, with an average of 12 defence mechanisms per genome. This is slightly higher than previous estimates from across a highly diverse panel of 21,364 bacterial genomes [41] and comparable to other plant pathogens such as *Ralstonia* and *Xanthomonas* isolates [41]. In sharp contrast to other bacterial species and pseudomonads [59,60], CRISPR-Cas does not appear to be an important phage defence strategy in the *P. syringae* species complex [61,62]. Instead, these bacteria encode restriction enzymes and abortive infection strategies primarily to potentially cope with phage predation. Further research should focus on the expression and function of these mechanisms during phage predation.

Despite a clear phylogenetic signal for phage defence mechanisms, we found an average of 4 prophage-like elements per genome across our panel of 587 genomes, comparable to a recent study on a collection of *P. syringae* isolates collected from apricot trees [58] and to other phytopathogens such as *Erwinia amylovora* [60]. Complete genomes had significantly more elements than draft assemblies, suggesting our numbers may underestimate prophage abundance. Notably, isolates with the highest number of prophage-like elements, defences, and effectors were host-associated, with phylogroup 1 showing the most elaborate defensomes and effectorome. This contrasts with phylogroup 10, which is highly pathogenic bacteria with significantly lower phage defences on average.

The widespread maintenance of prophages suggests they offer selective advantages despite their potential to kill their hosts. We observed a total of 83 unique prophages encoding a phage defence mechanism and/or an effector gene, potentially influencing their host’s interaction with the other phages and/or the plant host. Our findings suggest that lysogenic conversion appears most apparent in phylogroup 4, with a total of 19 different prophages carrying at least one effector gene (and/or defence mechanism, Fig. 4). Based on this observation, we can hypothesize that the virulence of isolates from phylogroup 4 is quite dependent on prophage-encoded effector genes. Indeed, a comprehensive study of the association between prophages and effector genes in * P. syringae* pv. *morsprunorum* isolates belonging to phylogroup 4 demonstrated the successful distribution of a prophage encoding HopAR1 from epiphytes within the phyllosphere community to pathogenic *P. syringae* pv. *morsprunorum* isolates [35]. Our taxonomical analysis revealed that effector-encoding phages were primarily lambdoid phages, similar to Shiga toxin-encoding lambdoid phages in *E. coli* [63]. Together with the observation that lambdoid phages may play a role in driving pathogen–host interactions in other bacterial species, our results raise the question of the overall importance of these phages in the dispersal of virulence factors among pathogens. Alongside the effector genes, many anti-phage mechanisms are encoded in the prophage genomes that defend the host [18,20, 64,67]. We found that the *Peduoviridae* or P2-like phages encoded phage defences*,* such as restriction enzymes and other phage defences that underlie superinfection exclusion (Figs4  5). Similarly, *E. coli* P2-like phages, together with P4-like satellites, carry diverse anti-phage systems, suggesting the importance of these phages in the establishment of the host’s phage defensome [68]. Based on these findings, we can hypothesize that this family of phages potentially drives the evolution of their hosts and interactions within the microbial community, in particular, the sensitivity of isolates to other phages within the viral community.

Altogether, our results suggest that specific clades of prophages may influence the evolution and ecology of their host by horizontally dispersing genetic cargo relating to interactions with host organisms and other phages. A recent analysis on a collection of * P. syringae* isolates collected from apricot trees demonstrated that prophage abundance exhibited a strong, non-linear relationship with bacterial susceptibility to phage infection [58]. Based on our results, lambdoid phages and members of the *Peduoviridae* appear to influence the virulence of their hosts across bacterial species, allowing us to hypothesize on the intimate relationship between these viruses and their hosts. Our analysis, however, strongly depends on the availability of genomes across the species complex of *P. syringae*, the quality of the genomes and the correct annotation of effector genes, defence mechanisms and prophage genomes. In addition, precise prophage boundaries in fragmented genomes are inherently uncertain and boundary miscalls can place bacterial genes adjacent to integration sites into prophage bins. Although we only focused on complete prophages in our analysis, the assignment of defence and effector genes to prophage genomes relies heavily on computational predictions. With reduced sequencing costs and upcoming advances in both long-read sequencing and the accuracy with which prophages are annotated by recent tools such as geNomad [69], we predict that the accuracy of analyses like this one will improve significantly in the foreseeable future. Access to higher quality, resolved contigs will enable us to capture other mobile genetic elements (MGEs) more efficiently, such as plasmids and integrative and conjugative elements (ICEs), and identify their role in shaping ecological interactions like bacterium–virus and bacterium–plant interactions, which we did not include in this study [70]. The availability of these data, and in particular from underrepresented phylogroups, will help us understand the complexity of prophage-mediated evolution across the entire species complex. Furthermore, as this study employs a comparative genomics approach, we have no insights into the actual transcription of the defence mechanisms, nor the effector proteins. Future endeavours should focus on the *in situ* expression of these genes to unravel how they steer the evolution of and interaction between the phages, bacteria and ultimately the plant and experimentally validate the computational findings from this study. Similarly, future research should focus on the activity of the prophages described in this analysis and their ability to distribute genes from one host to the next. As mentioned earlier, Hulin *et al.* have presented the first endeavours to demonstrate the distribution of virulence genes between *P. syringae* isolates by prophages [35]. Yet, knowledge on the expansion of genes that are distributed by prophages among other genomic parasites within microbial communities is lacking. As such, our analysis, through comparative genomics, provides one of the first critical steps to understand which genes are distributed and ultimately play a role as genomic parasites in the evolutionary trajectory of their host.

## Conclusion

As genomic parasites of bacteria, prophages are an important part of the bacterial mobilome, but can also kill their host cells under environmental stress. The fact that these Trojan horses are widespread in bacterial genomes implies an evolutionary advantage for maintaining these parasites in the genome. In our study, we demonstrated the role of specific viral taxa in driving the effectorome and phage defensomes in certain phylogroups within the *P. syringae* species complex, potentially steering bacterium–plant and bacterium–virus interactions. We further provide statistical evidence that host-associated isolates have more elaborate phage defensomes, effectoromes and prophage-like elements incorporated in their genomes. This raises questions on the role of these viruses in the dispersal of these genes within the broader host-associated microbial community, as well as the frequency of temperate phage infections within the microbiome and how these viruses are shaping the evolutionary trajectory of pathogens and other members within the microbial community. Our study also provides insights into the role of phage defences, characterized by an incredible diversity and distribution in bacterial genomes, in phage–phage competition alongside bacterial defence against phage infection.

## Supplementary material

10.1099/mgen.0.001711 Supplementary Material 1.

10.1099/mgen.0.001711 Supplementary Material 2.

## Abbreviations

ANOSIM: analysis of similarity
ICE: Integrative and Conjugative Element
MGE: Mobile Genetic Element
