# Erratum: Comparative genomics reveals high prophage diversity and horizontal gene transfer of effectors and phage defence systems in the Pseudomonas syringae complex

**DOI:** 10.1099/mgen.0.001761

**Published:** 2026-06-18

**Authors:** Dominique Holtappels, George E.J. Rickus, Túlio Morgan, Rafael R. de Rezende, Britt Koskella, Poliane Alfenas-Zerbini

**Affiliations:** 1 Plant Pathology and Plant-Microbe Biology section, School of Integrative Plant Science, Cornell University, Cornell AgriTech, Geneva, NY, USA; 2 Department of Integrative Biology, University of California, Berkeley, CA, USA; 3 Departamento de Microbiologia, Instituto de Biotecnologia Aplicada à Agropecuária, Viçosa 36570-900, MG, Brazil; 4 Chan-Zuckerberg Biohub, San Francisco, CA, USA

In the published version of this article, the legends for Figs2  3 were incorrectly switched. The correct figure legends are provided below. The published article has been updated accordingly.

**Fig. 2.**
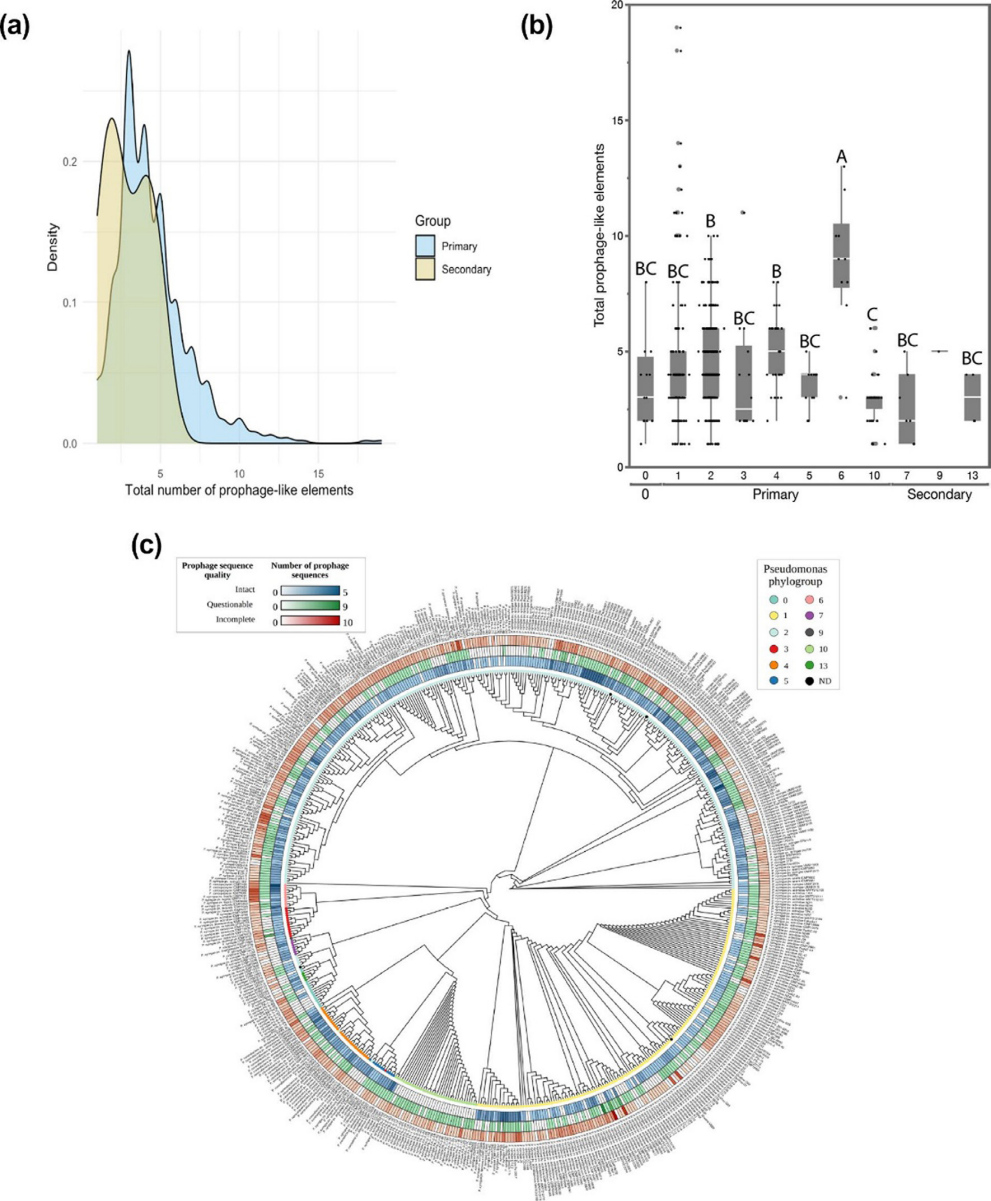
Number of prophage-like elements encoded in the P. syringae species complex. (a) Distribution of primary (blue) and secondary (yellow) phylogroups and the total number of prophage-like elements encoded with significantly more prophage-like elements encoded in the primary phylogroups (Student’s t-test, P-value=0.0017). (b) Quantile box plots of the total number of prophage-like elements per individual phylogroup. Based on a Tukey –Kramer test with corrections for multiple comparisons, three groups of significance were identified with phylogroups 6 on average the highest number of prophage-like elements per genome as summarized with connecting letters indicating the groups of significance (A, B, and C). (c) Phylogenetic tree of 587 Pseudomonas isolates. The ‘'ggtree’' package in the R environment was used for tree visualization ignoring branch lengths. Heatmaps were added as concentric rings at the tree tips, representing the quality and quantity of prophages detected in each P. syringae genome by PHASTER.

**Fig. 3.**
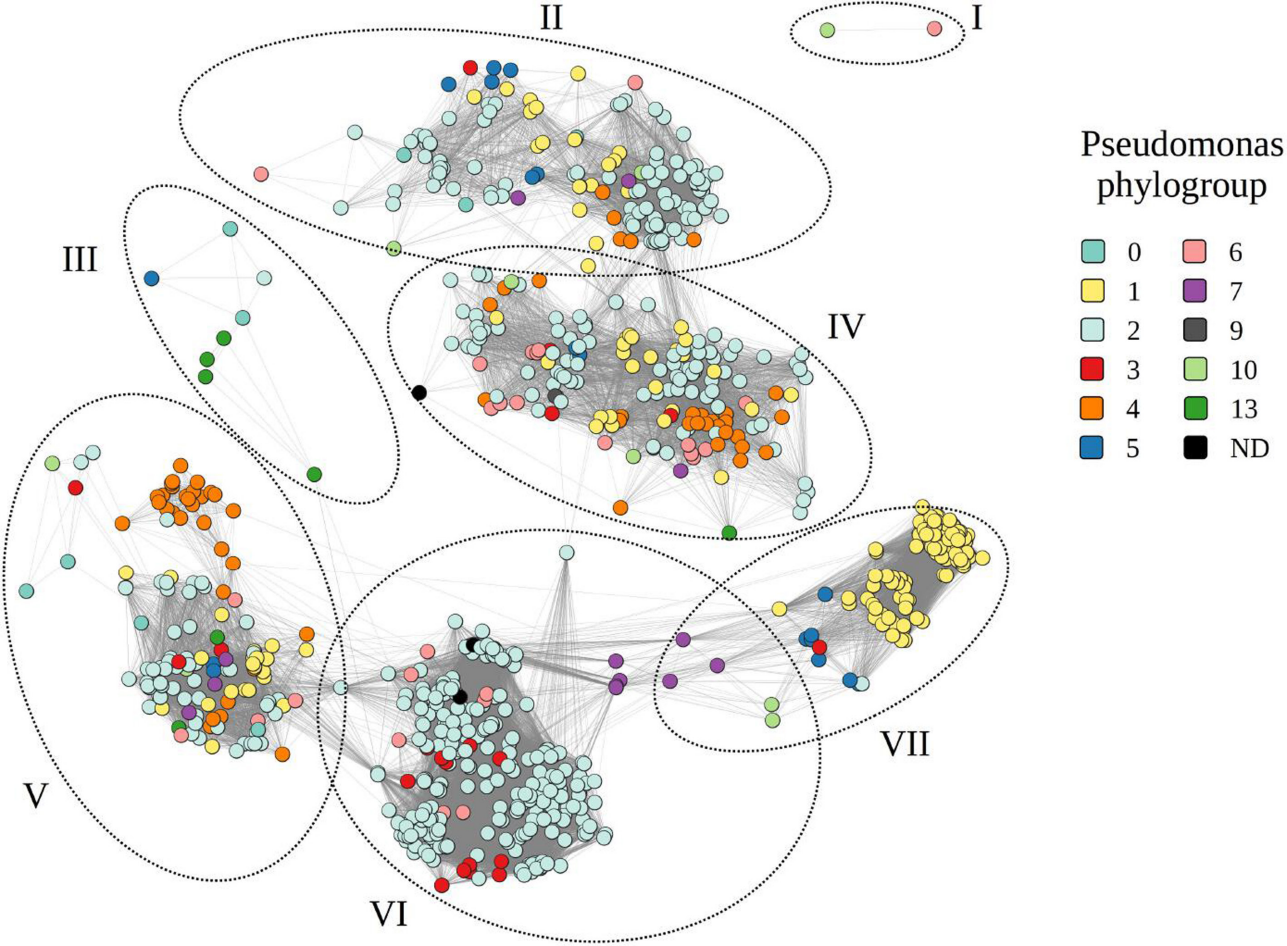
Gene sharing network between the different prophage genomes annotated in the P. syringae species complex. The nodes are coloredcoloured along the phylogroup (PG0, teal,; PG1, yellow,; PG2, light blue;, PG3, red;, PG4, orange;, PG5, blue;, PG6, pink;, PG7, purple;, PG9, grey;, PG10, light green;, PG13, dark green). Every connection represents a similarity to another predicted viral sequence. The different clusters are outlined and numbered (I, II, III, IV, V, VI, VII).

The Microbiology Society apologises for any inconvenience caused.

