# Supplementary material for: Comparative genomics reveals high prophage diversity and horizontal gene transfer of effectors and phage defence systems in the Pseudomonas syringae complex
Source: Microb Genom. 2026 May 18;12(5):001711. doi: 10.1099/mgen.0.001711 (PMC13182921; doi:10.1099/mgen.0.001711)
Supplement: Supplementary Material 2. [file mgen-12-01711-s002.pdf]

Supplementary Figures

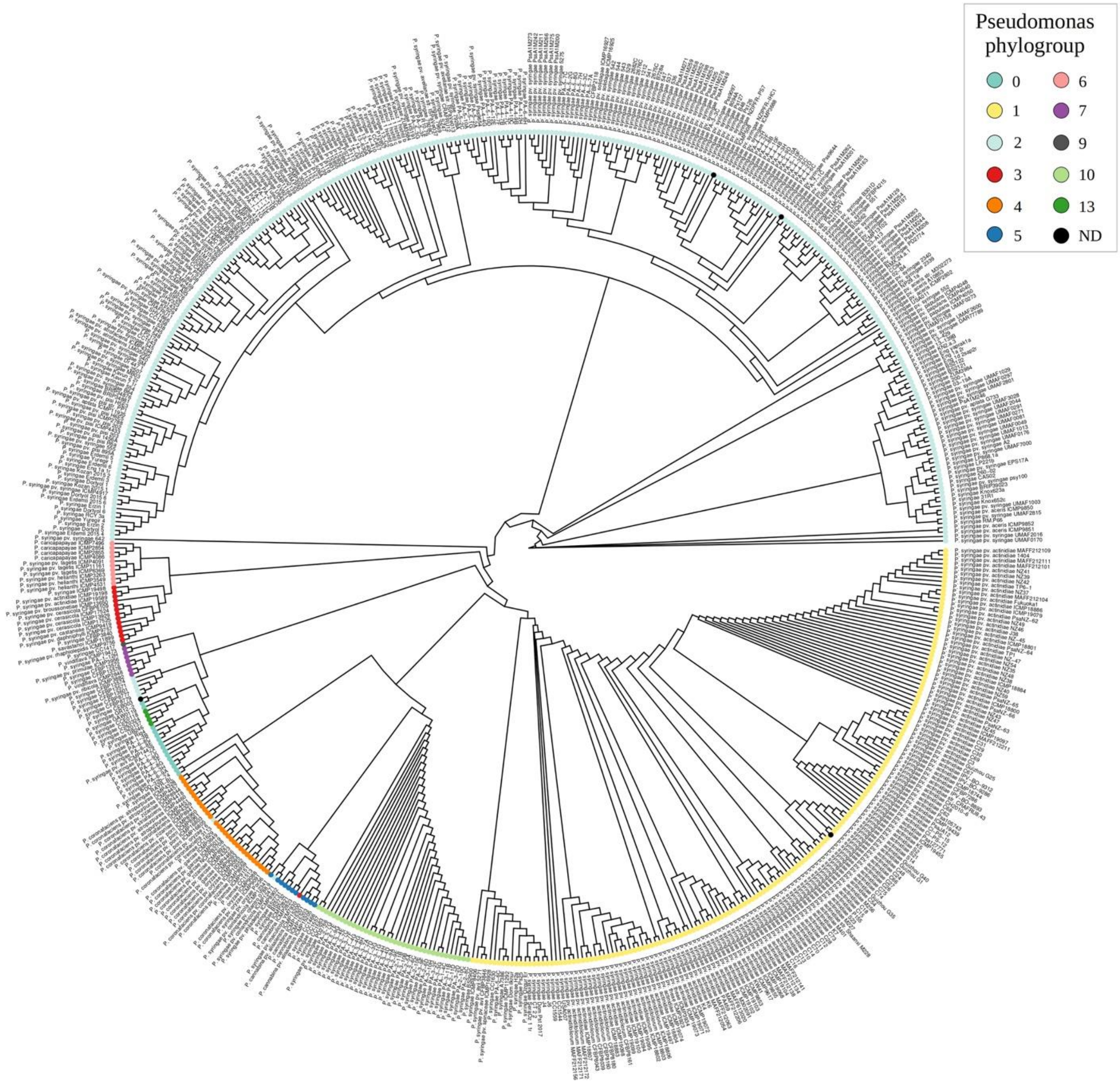

Supplementary Figure 1 Phylogenetic tree of 587 Pseudomonas isolates based on the core genome as calculated using PpanGGOLin. The 'ggtree' package in the R environment was used for tree visualization, ignoring branch lengths. Pseudomonas phylogroup information was added as colored circles at the tree tips.

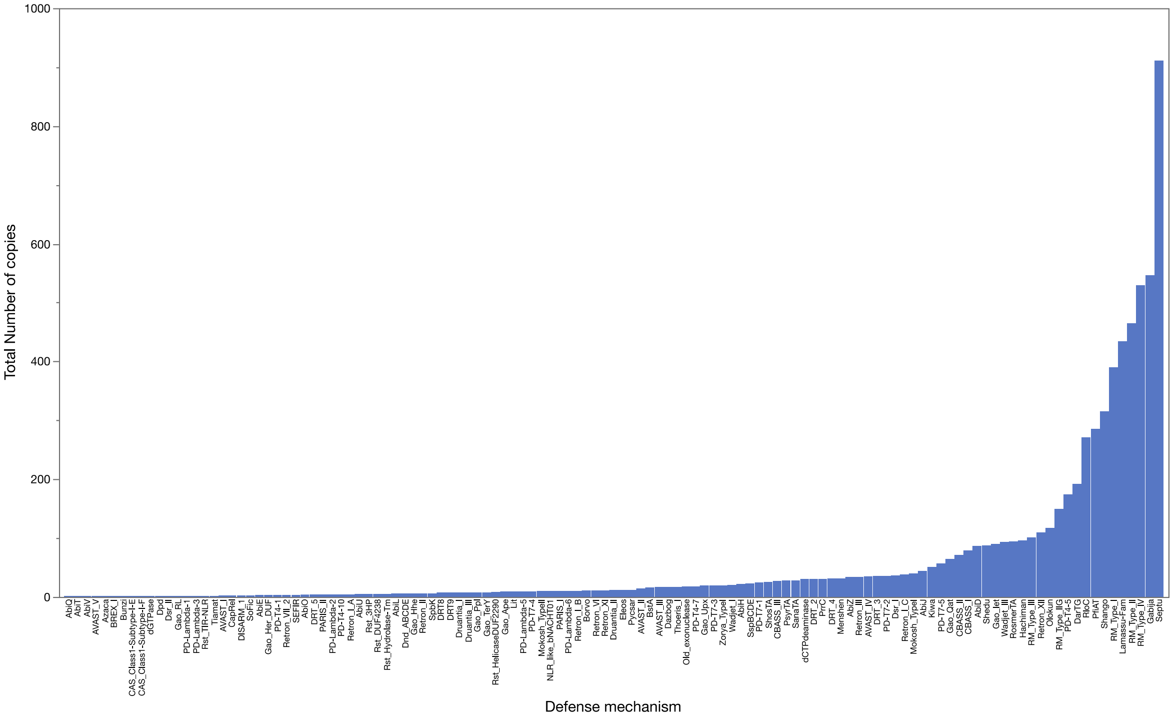

Supplementary Figure 2 Overview of the full phage defensome of the *P. syringae* species complex with septu and restriction enzymes the most abundant phage defenses.

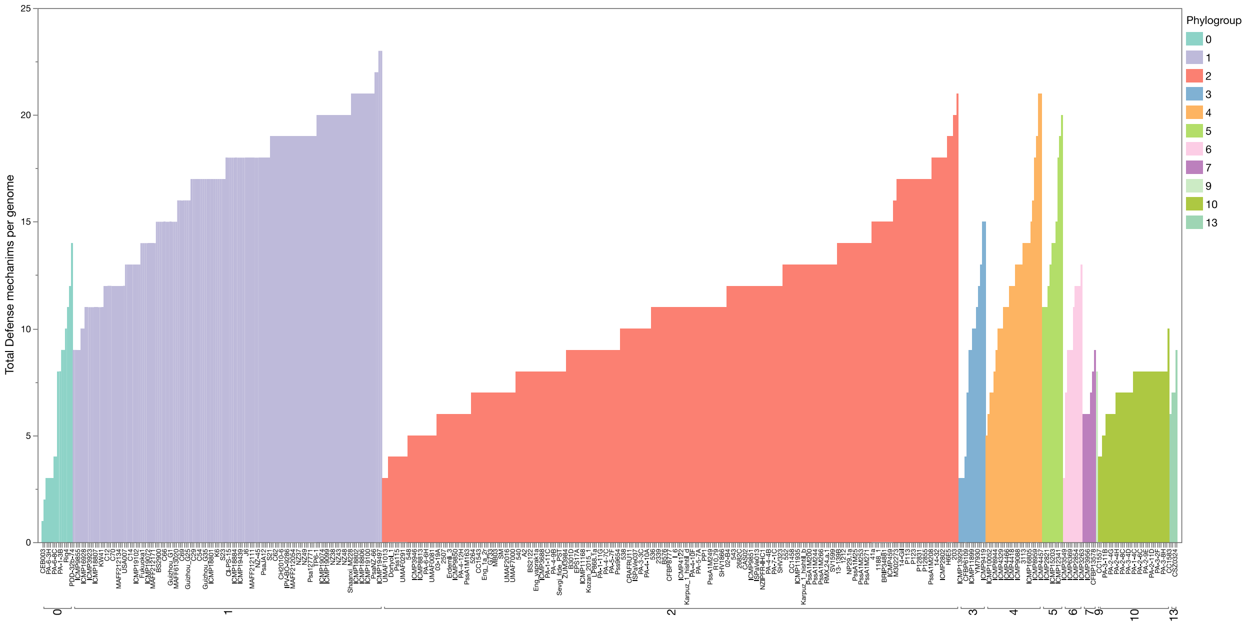

Supplementary Figure 3 Overview of the number of defenses encoded by the different isolates organized per phylogroup. In cyan PG0, purple PG1, orange PG2, blue PG3, yellow PG4, light green PG5, pink PG6, violet PG7, green PG10, and teal PG13.

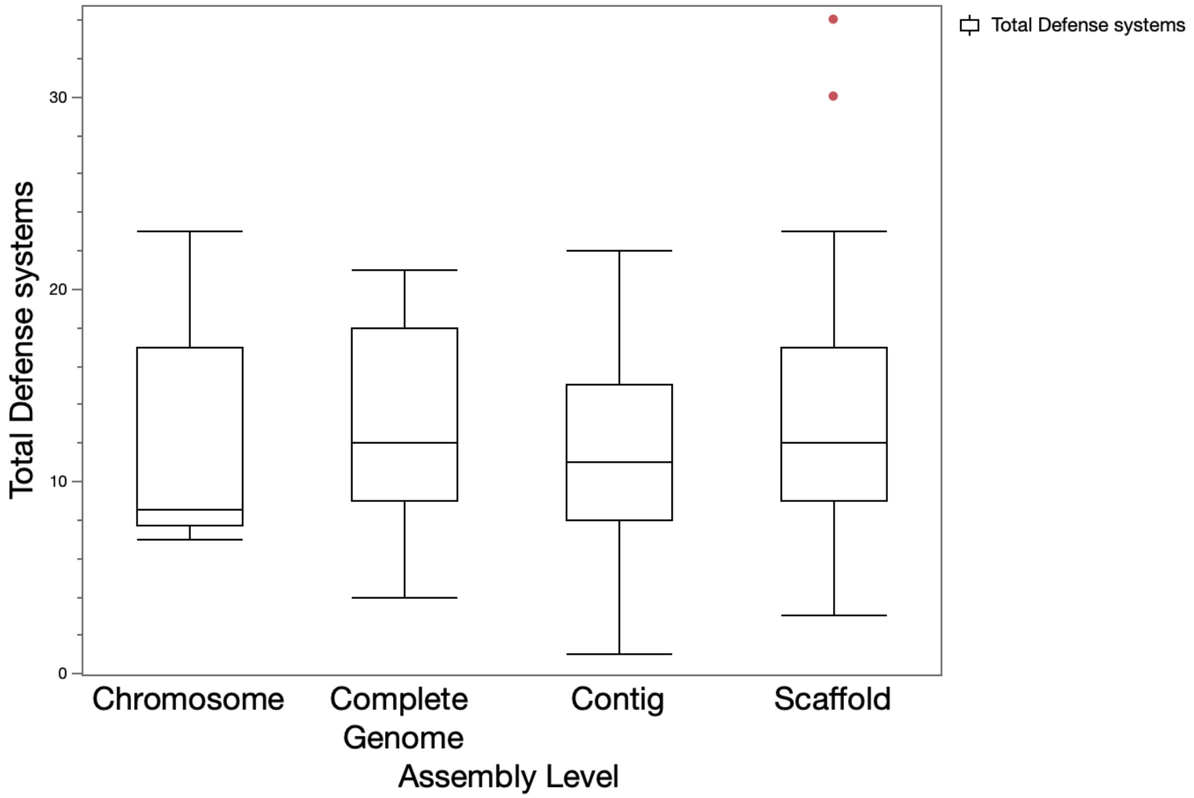

Supplementary Figure 4 The number of phage defense mechanisms annotated per genome as expressed per assembly level as extacted from NCBI. There is no significant difference between the groups as determined by ANOVA (p-value = 0.2).

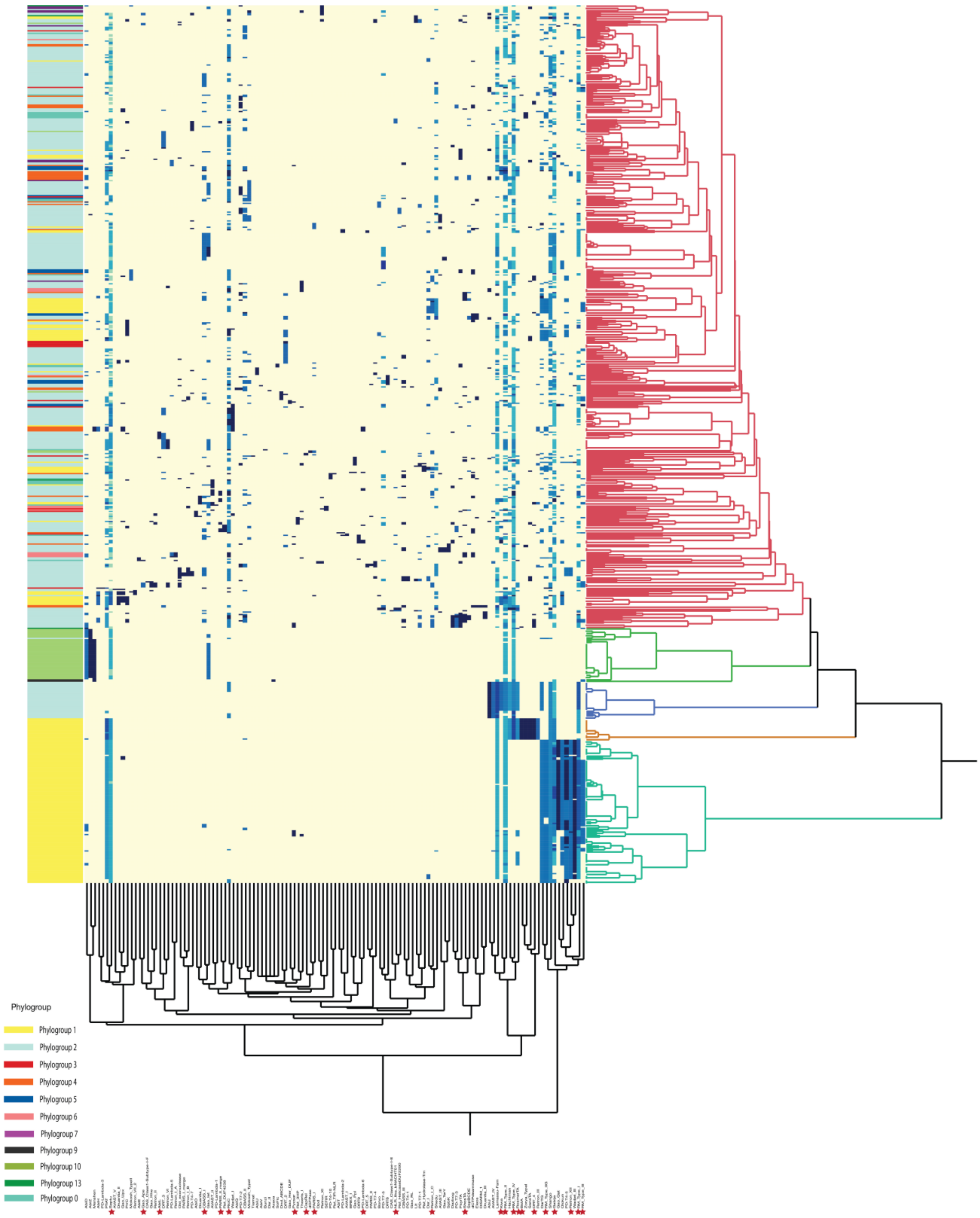

Supplementary Figure 5 Hierarchical clustering of the absence/presence matrix of the different phage defense mechanisms (x-axes) annotated in the different genomes (y-axes). Phylogroups are indicated on the left-hand panel: PG1 yellow, PG2 light blue, PG3 red, PG4 orange, PG5 dark blue, PG6 pink, PG7 purple, PG9 grey, PG10 light green, PG11 dark green and PG0 teal. The

different clusters as determined by the Ward hierarchical clustering method are shown on the right-hand (red, green, blue, orange, and teal). Five clusters are calculated and color coded. Defense mechanisms that were annotated within prophage genomes are indicated by a red star.

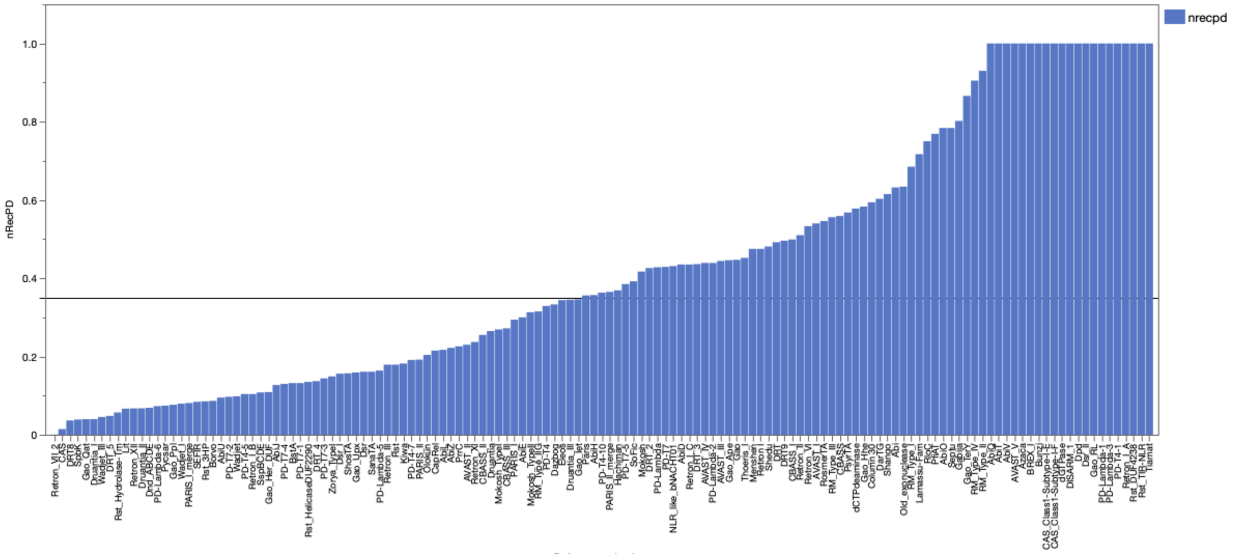

Supplementary Figure 6 nRecPD values for the different phage defense mechanism calculated based on the phylogenetic distance as determined by a core genome alignment of the different isolates. The threshold of 0.35 indicates mechanisms that are likely to be distributed through horizontal gene transfer (value closer to 0) or vertically inherited (value closer to 1).

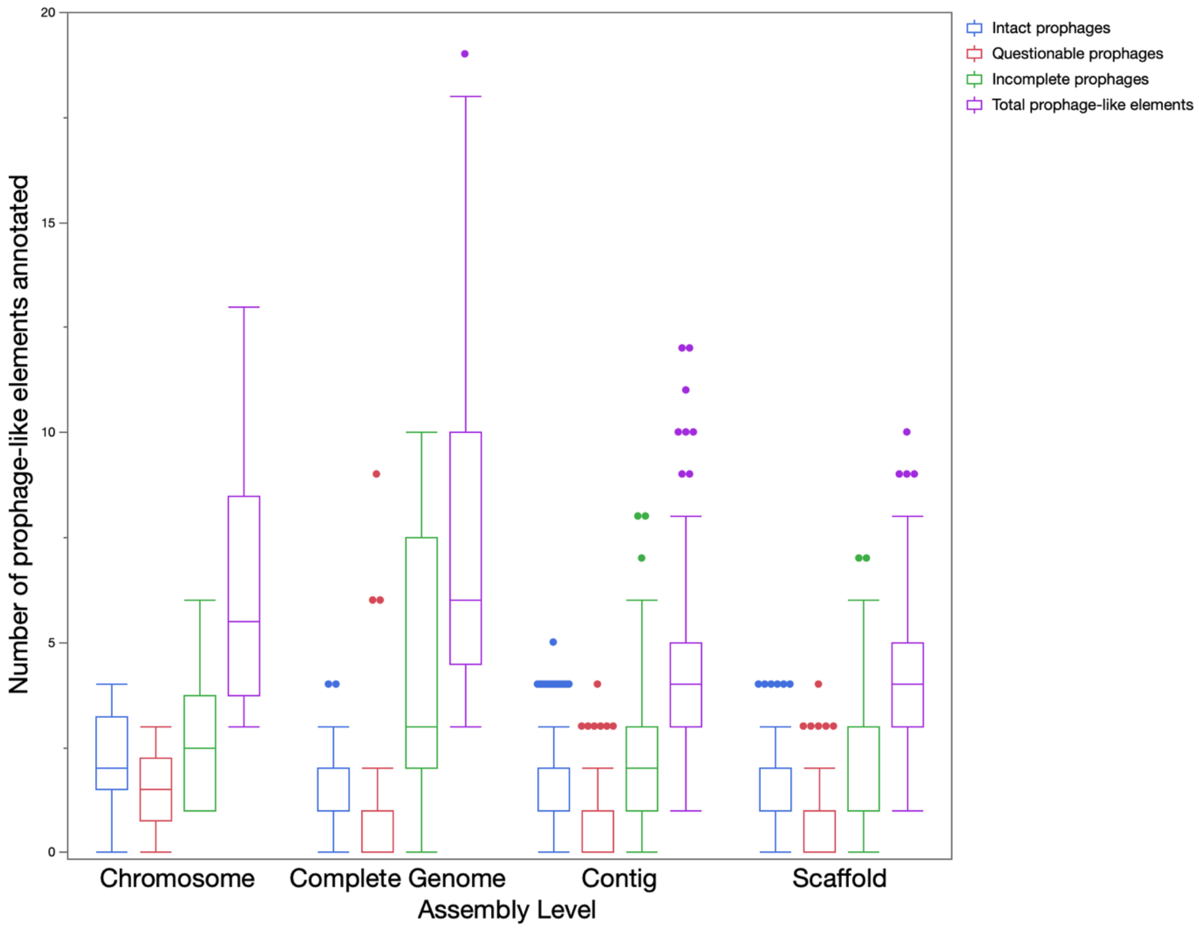

Supplementary Figure 7 Number of prophage-like elements annotated in a given genome per assembly level. There are significant differences for total prophage-like elements (purple, ANOVA p-value = 0.001), questionable prophages (red, ANOVA p-value = 0.02), incomplete prophages (green, ANOVA p-value = 0.001), and intact prophages (blue, ANOVA p-value = 0.001).

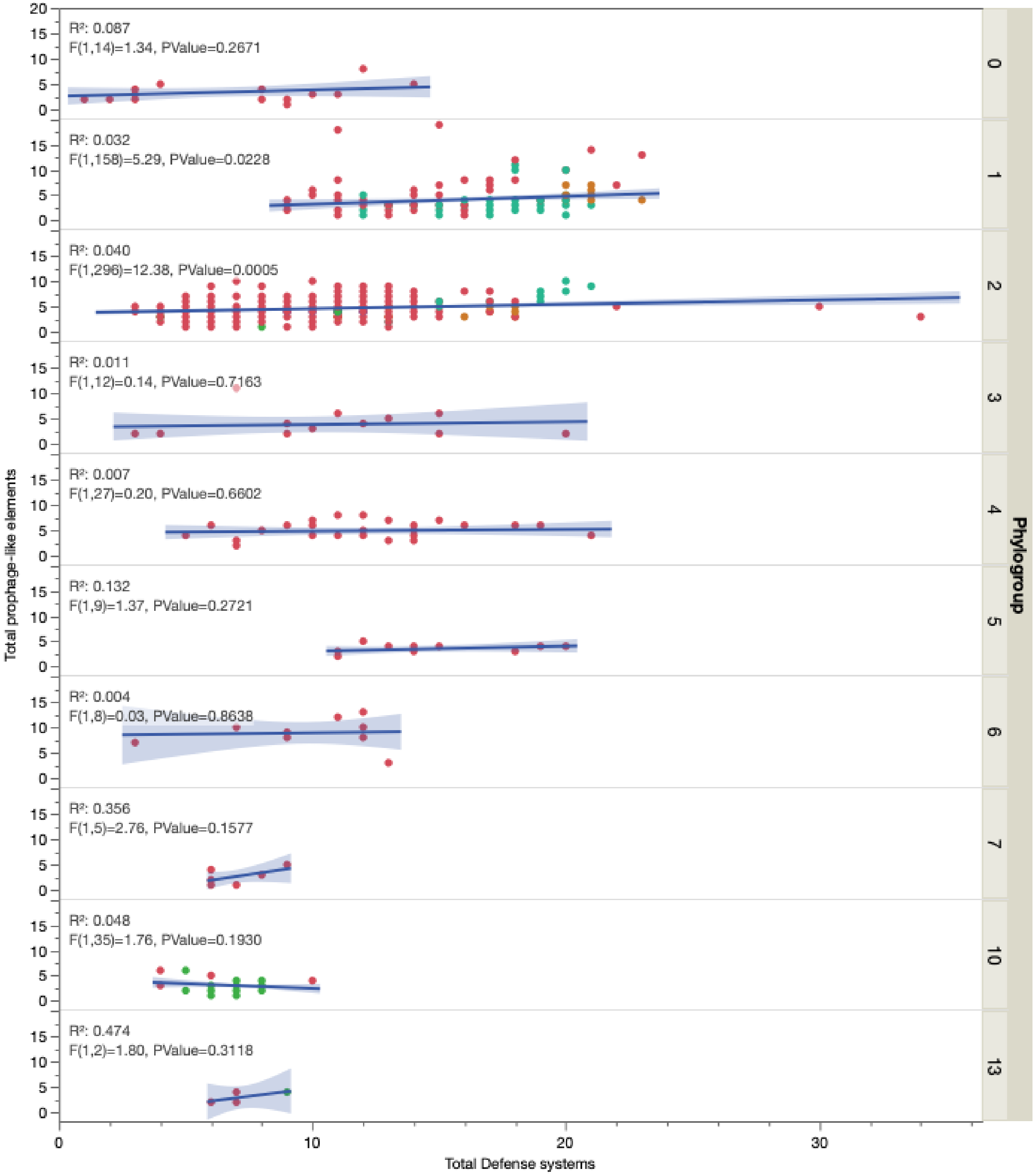

Supplementary Figure 8 Linear regression of the total number of prophage-like elements encoded in function of the total number of phage defenses per isolate organized per phylogroup. A rather low R^2 value indicates a weak correlation between the two variables,

suggesting that the total number of phage defenses is a poor predictor for the number of genomic parasites in the genome.

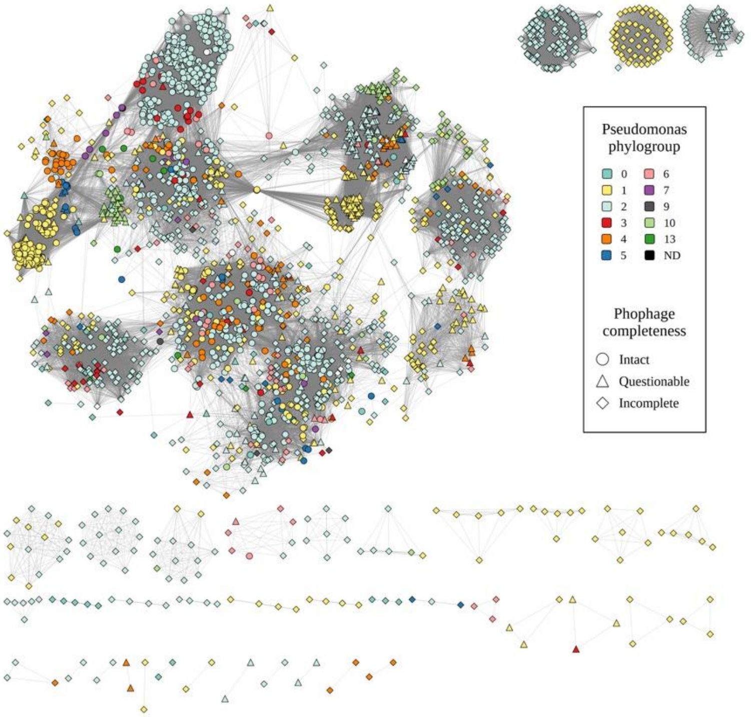

Supplementary Figure 9 Gene sharing network between all prophage-like elements annotated in the *P. syringae* species complex. The nodes are colored along the phylogroup. Complete prophage genomes are depicted as circles, questionable prophages as triangles, and incomplete prophages as diamonds.

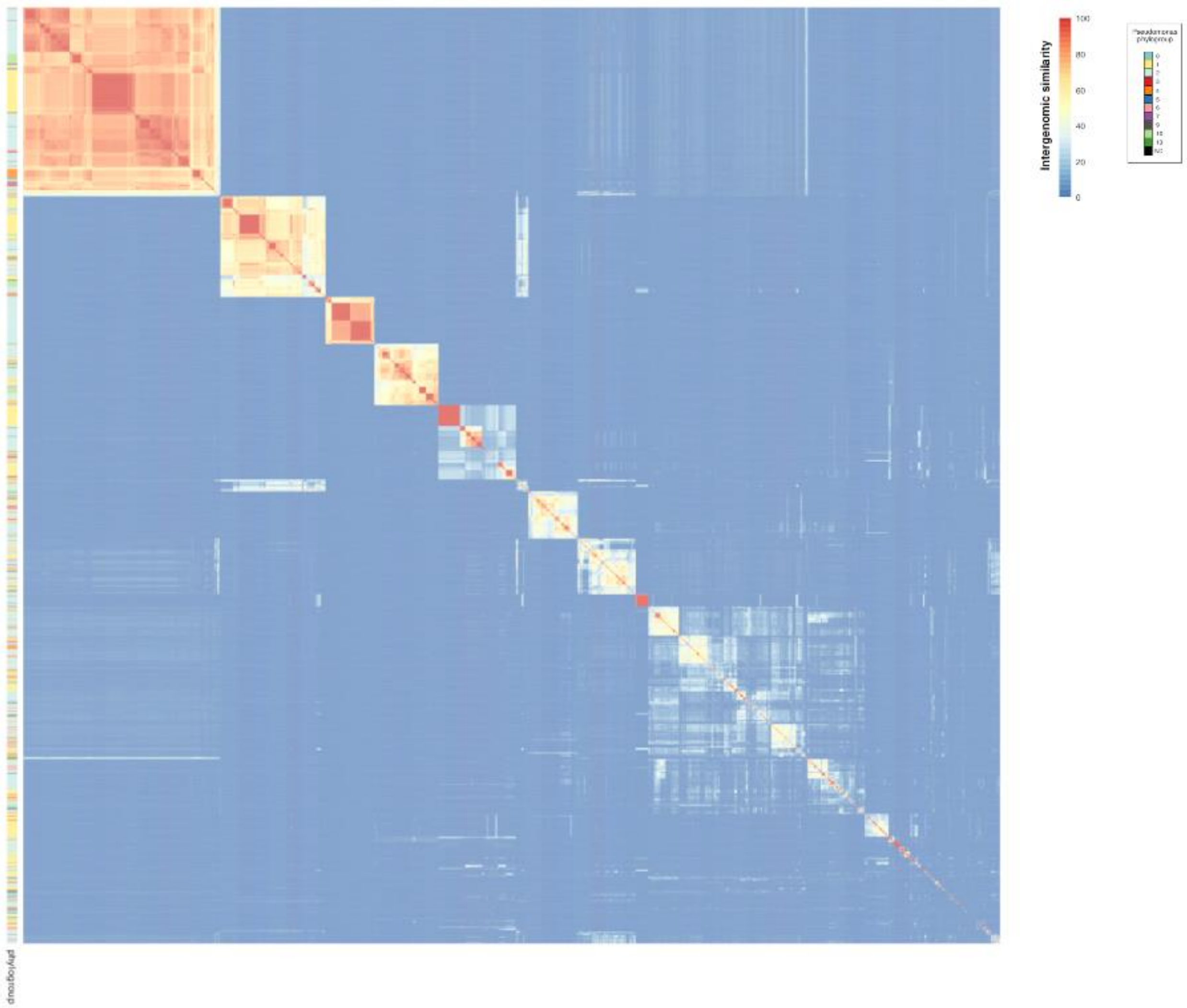

Supplementary Figure 10 Intergenomic similarity (VIRIDIC) of all prophage-like elements encoded in the *P. syringae* species complex. The left hand bar color codes the phylogroups in which the prophage-like element was annotated.

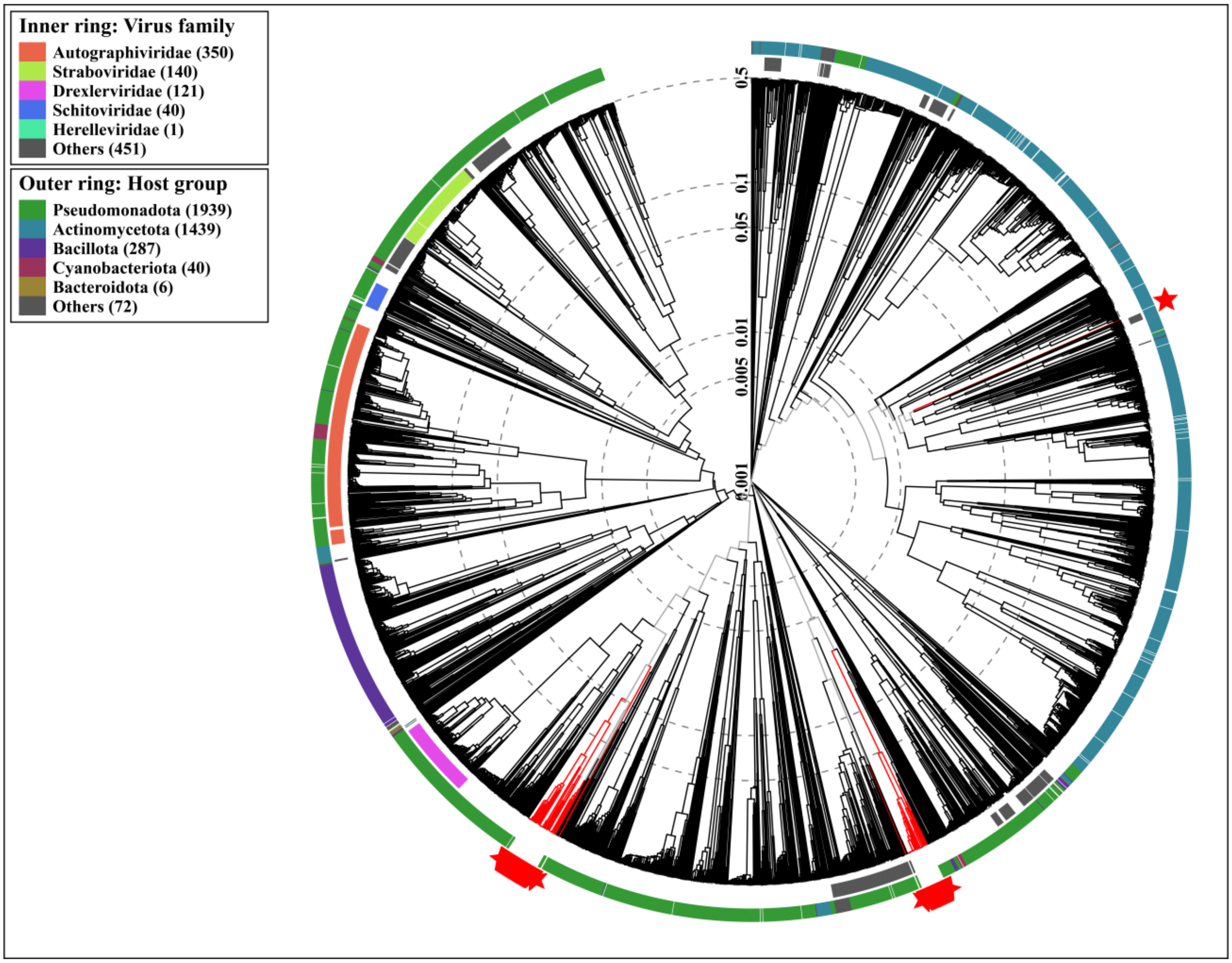

Supplementary Figure 11 VipTree analysis of the prophage genomes encoding effector and/or phage defenses. Genomes from this analysis are indicated by the red stars. Two different clades can be distinguished and one orphan phage.

Supplementary Table 1 Overview of the different genomes used in this study
